# Undernutrition and associated factors among children aged 6-59 months in East Belesa District, northwest Ethiopia: a community based cross-sectional study

**DOI:** 10.1186/s12889-016-3180-0

**Published:** 2016-06-13

**Authors:** Wagaye Fentahun, Mamo Wubshet, Amare Tariku

**Affiliations:** Child Health and Nutrition Core Process, Gondar Zuria District Health Office, North Gondar Administrative Zone, Gondar, Ethiopia; Department of Environmental and Occupational Health and Safety, Institute of Public Health, University of Gondar, Gondar, Ethiopia; Department of Human Nutrition, Institute of Public Health, University of Gondar, Gondar, Ethiopia

**Keywords:** Children under five years, Undernutrition, Stunting, Wasting, Northwest Ethiopia

## Abstract

**Background:**

Undernutrition remains the major public health concern in Ethiopia and continues as the underlying cause of child mortality. However, there is a scarcity of information on the magnitude and determinant factors of undernutrition. Therefore, this study aimed to assess the prevalence of undernutrition and associated factors among children aged 6–59 months in East Belesa District, northwest Ethiopia.

**Methods:**

A community-based cross-sectional study was conducted from April to May, 2014. A multistage stratified sampling technique was used to select 633 study participants. A structured interviewer-administered questionnaire was used to collect data. In order to identify factors associated with undernutrition (stunting and wasting) a multivariate logistic regression analysis was employed. The Adjusted Odds Ratio (AOR) with a 95 % Confidence Interval (CI) was computed to show the strength of the association. In the multivariate analysis, variables with a p-value of <0.05 were considered as statistically significant.

**Results:**

In this study, about 57.7 and 16 % of the children were stunted and wasted, respectively. The odds of stunting were higher in children born to mothers who gave their first birth before 15 years of age (AOR = 2.4; 95 % CI: 1.19, 5.09) and gave prelacteal feeding to their child (AOR = 1.83; 95 % CI: 1.28, 2.61). However, lower odds of stunting were observed among children aged 36–47 months (AOR = 0.41; 95 % CI: 0.22, 0.78) and had higher family monthly income, Et. Br. 750–1000, (AOR = 0.61; 95 % CI: 0.39, 0.92). Moreover, the odds of wasting were higher among children who received butter as prelacteal food (AOR = 2.32; 95 % CI:1.82, 5.31).

**Conclusion:**

Child undernutrition is a critical public health problem in the study area. Advanced age of children (36–47 months) and higher family monthly income were inversely associated with stunting. However, higher odds of stunting were observed among children whose mothers delivered their first child before 15 years of age, and gave their children prelacteal feeding. Thus, delaying the first pregnancy and reducing prelacteal feeding is of a paramount significance in reducing the burden of undernutrition.

## Background

Nutritional status of children is an important outcome measure of their health status [1]. Child undernutrition (stunting and wasting) which is highly prevalent in low and middle income countries is crippling global economic growth [2, 3]. Wasting is a health condition that reflects acute nutritional deficit, while stunting is a measure of linear growth retardation showing the cumulative effect of chronic food deprivation [1]. Globally, about one quarter (26 %), and nearly one-tenth (8 %) of children are stunted and wasted, respectively [3]. A considerable burden (80 %) of global undernutrition is observed in developing countries where most of the children live in substandard and insanitary conditions [4]. Asia alone bears 42 % of the stunting and 70 % of the wasting. Likewise, in Africa, the overall prevalence of stunting is 47 % [5]; it is 42 % in East Africa [3].

In Ethiopia, the burden of child undernutrition has persisted as a severe public health problem for decades. The 2011 Demographic and Health Survey (DHS) Report, according to which about 44 and 10 % of the children were stunted and wasted, respectively, confirmed the public health significance of undernutrition [1]. Furthermore, because of the difference in socio-economic and ecological characteristics, the magnitude of undernutrition exhibited regional variations ranging from 24 to 67 % for stunting and 11–17 % for wasting [6–8].

Globally, undernutrition is widely considered as the underlying cause for 3.1 million child deaths, 45 % of all causes of mortality [3]. The risk of mortality worsens, especially when a child suffers from stunting and wasting at the same time [9]. Stunting correlates to poor cognition and low school performance and a higher risk of developing nutrition-related chronic diseases in later life [2, 4, 10–12]. In Ethiopia, though there has been a significant achievement in reducing the overall child mortality, still 88 children per 1000 live births die before celebrating their fifth birth day [1], and undernutrition remains the underlying cause for 28 % of child mortality. Similarly, it was estimated to cause loss of Et.Br. 55.5 billion per annum, which is equivalent to 16.5 % of the country’s Gross Domestic Product (GDP). Sixteen percent of all failures in primary schools are also associated with stunting [13].

Child undernutrition is the consequence of complex interactions of various factors mainly related to socio-economic, feeding pattern, health care, and environmental factors. Previous reports from different countries claimed that poor feeding practices, rural residence, maternal and paternal illiteracy, advanced maternal age, poor household economic status, non-attendance of antenatal care, poor access to safe water, unavailability of toilet facility, closed birth interval, and a large number of siblings are factors significantly associated with undernutrition [7, 14–27].

Mindful of the adverse consequences of undernutrition, Ethiopia has set a target to reduce stunting from 44 to 30 %, and wasting from 10 to 3 % [28]. To this end, the country designed different nutritional strategies and programs [28, 29] though no significant improvements have yet been attained [1]. Furthermore, studies carried out in the drought prone areas of the country noted the heaviest burden of undernutrition compared to the national average [8, 30]. Likewise, East Belesa District, the current study area, as one of the drought prone areas needs further emphasis and investigation of the problem. However, there is a scarcity of information about the magnitude and the determinant factors of undernutrition in northwest Ethiopia, particularly East Belesa District. Thus, this study aimed to assess the prevalence of undernutrition (stunting and wasting) and associated factors among children aged 6–59 months in the area in order to fill the knowledge gap.

## Methods

### Study design and setting

A community-based cross-sectional study was conducted from April to May 2014 in East Belesa District, northwest Ethiopia. The district has 22 kebeles *(smallest administrative units in Ethiopia),* 20 of which are rural. Out of the total 111,687 inhabitants, 14.6 % are children under five years of age. The district is situated at an altitude ranging from 1496 to 2000 m above sea-level. About 90% of the district is desert *(kola)* with a minimal annual rain fall leading to frequent drought and famine. In spite of the climatic condition, the people depend on agriculture and cultivate cereals, like teff, beans, sorghum, and wheat.

### Sample size and sampling procedures

Mother-child pairs who lived in East Belesa District for at least six months were eligible for the study. Sample size was determined using the single population proportion formula with the following assumptions: the prevalence of stunting among children aged 6–59 months was 52 % in Amhara Region [2], a 95 % level of confidence, 5 % margin of error, a design effect of 1.5, and a ten percent contingency for non-response rate. Finally, the minimum sample size required for the study (633) was obtained. A multi-stage stratified sampling, followed by a systematic random sampling technique was employed to reach the study subjects. The kebeles were stratified into urban and rural settlements. Out of the 22 kebeles, 5 (one urban and four rural) were selected by the lottery method. The total number of households with children aged 6–59 months (2735) was accessed from the Health Extension Workers Housing Registration. Then the number of households sampled from the selected kebeles was determined proportionally. In addition, the sampling interval was calculated, and households with eligible children were selected using a systematic random sampling technique. For households with more than one children, a child was selected using the lottery method. When mother-child pairs were not available at the time of data collection, another visit was made. When that failed, the nearby household was considered.

### Data collection instruments and procedures

Data were collected using a structured interviewer-administered questionnaire. To maintain consistency, the questionnaire was first translated from English to Amharic (the native language of the study area) and was retranslated to English by professional translators. The questionnaire was designed with four major sections, like socio-demographic and economic characteristics, child feeding practices, maternal and child health care, and household related factors. The tool was piloted on 32 mother-child pairs outside the study area. During the pre-test, the acceptability and applicability of the tools and procedures was evaluated. Interview was conducted with mothers. Ten clinical nurses collected the data under the supervision of two health officers. The data collectors and supervisors were given a two-day intensive training ahead of time.

### Anthropometric measurements

Child weight was measured to the nearest 0.1 kg by the seca beam balance (German, Serial No. 5755086138219) with graduation of 0.1 kg and a measuring range of up to 25 kg. Weight was taken with light clothing and no shoes. Instrument calibration was done before weighing each child. Furthermore, the weighing scale was checked daily against the standard weight for accuracy. Height was measured using the seca vertical height scale (German, Serial No. 0123) standing upright in the middle of the board. The child’s head, shoulders, buttock, knees, and heels touch the vertical board. The length of a child (aged 6–23 months) was measured using a horizontal wooden length board in recumbent position, and read to the nearest 0.1 cm. To check edema, normal thumb pressure was applied on both feet for three seconds. The data collectors checked whether a shallow print remained on both feet or not when the thumb was lifted.

### Data analysis

Data were entered into the EPI-info version 3.5.3 statistical software, and analyzed using SPSS version 20 statistical package. Nutrition related data (sex, age, height, weight, and edema status) were transferred to the ENA/SMART software version 2012. The Z-scores of indices, Weight-for-Hight Z-score (WHZ) and Hight-for-Age Z-score (HAZ) were calculated and compared using the World Health Organization (WHO) Multicenter Growth Reference Standard. A child whose HAZ less than −2 Standard Deviation (SD) from the reference population was defined as stunted, while a child with WHZ less than −2 SD from the reference population was classified as wasted [31]. Descriptive statistics, including frequencies and proportions were used to summarize the variables. Bivariate analysis was done individually for all independent variables with stunting and wasting. Variables with a p-values of <0.2 in the bivariate analysis were entered to a multivariate logistic regression analysis to identify the independent determinants of stunting and wasting separately. Both the Crude Odds Ratio (COR) and the Adjusted Odds Ratio (AOR) with a corresponding 95 % Confidence Interval (CI) were computed to show the strength of the association. In the multivariate logistic regression analysis, variables with a p-value of <0.05 were considered as statistically significant.

## Results

Six hundred thirty-three mother-child pairs were included in the study. The mean (±SD) age of the children was 26.5(±14.4) months, and one-fourth (28.1 %) were found in the age range of 12–23 months. Most (87.7 %) of the mothers were uneducated; about 94.9 % were housewives (Table 1).

**Table 1:** Socio-demographic and economic characteristics of the children and their parents, East Belesa District, northwest Ethiopia, 2014 (*N* = 633)

Variables	Frequency	Percent
Child sex		
Male	339	53.6
Female	294	46.4
Child age in month		
6–11	107	16.9
12–23	178	28.1
24–35	138	21.8
36–47	118	18.6
48–59	92	14.6
Maternal age		
15–19	31	4.9
20–29	335	52.9
30–51	267	42.2
Maternal age at first birth		
<15	63	10.0
15–19	444	70.1
20–29	126	19.9
Head of HH^a^		
Male	585	92.4
Female	48	7.6
Marital status		
Married	585	92.4
Single	17	2.7
Other^b^	31	4.9
Religion		
Orthodox	617	97.5
Muslim	16	2.5
Ethnicity		
Amhara	622	98.3
Agew	11	1.7
Family size		
<5	375	59.2
≥5	258	40.8
Number of children ever born		
1–3	372	58.8
4–6	226	35.7
>6	35	5.5
Number of under five children		
1	383	60.5
2–3	250	39.5
Maternal education		
No education	555	87.7
Informal education (can read and write)	43	6.8
Formal education^c^	35	5.5
Paternal education		
No education	303	50.2
Informal education (can read and write)	183	30.3
Formal education^c^	118	19.5
Maternal occupation		
Housewife	601	94.9
Others^d^	32	5.1
Paternal occupation		
Farmer	580	96.1
Other^d^	24	3.9
Monthly income (in Et. Br.)		
<750	456	72.0
750–1000	134	21.2
>1000	43	6.8
Decision making on use of money		
Individually^e^	483	76.3
Jointly	150	23.7
Livestock ownership		
Yes	521	82.3
No	112	17.7
HH supported by safety net program		
Yes	81	12.8
No	552	87.2

^a^Household

^b^Widowed and divorced

^c^Primary, secondary, and above

^d^Student, merchant and government employee

^e^Either by the husband or the wife

Nearly two-thirds (58.7 %) of the children were given prelacteal food; for 72.6 % the prelacteal food was butter. Moreover, half (55.5 %) of the children were initiated to complementary feeding at the right time, the sixth month of age. Two-thirds (67.8 %) of the children took vitamin-A supplementation in the past six months. About 26.1 and 18.5 % had history of diarrheal morbidity and fever, respectively, in the past two weeks preceding the survey. One-quarter (28.8 %) of the mothers had antenatal care visits for the index child (Table 2).

**Table 2:** Feeding practice and health care related characteristics of mother-child pairs, East Belesa District, northwest Ethiopia, 2014

Variables	Frequency	Percent
Initiation of BF^a^		
Within one hour	458	72.4
1–24 h	156	24.6
After 24 h	19	3.0
Prelacteal feeding		
Yes	372	58.7
No	261	41.3
Type of prelacteal food given (372)		
Water	102	27.4
Butter	270	72.6
Feeding colostrum		
Yes	410	64.8
No	223	35.2
Initiation to complementary feeding (*n* = 593)		
Before sixth month	105	17.7
At sixth month	329	55.5
After sixth month	159	26.8
Complementary food offered in the last 24 h		
Yes	593	93.7
No	40	6.3
Frequency of feeding (*n* = 593)		
<3 times	336	56.7
3 times	152	25.6
>3 times	105	17.7
Method of feeding		
Bottle	19	3.4
Cup	284	49.9
Spoon	73	12.8
Hand	193	33.9
Duration of BF		
<12 months	155	24.5
12–24 months	223	35.2
>24 months	255	40.3
Antenatal care follow-up		
Yes	182	28.8
No	451	71.2
Extra food during pregnancy or lactation		
Yes	200	31.6
No	433	68.4
Child vitamin-A supplementation in the past six months		
Yes	429	67.8
No	204	32.2
Diarrheal morbidity in the past two 2 weeks		
Yes	165	26.1
No	465	73.9
Fever in the past two 2 weeks		
Yes	117	18.5
No	516	81.5
ARI^b^ in the past two 2 weeks		
Yes	96	15.2
No	537	84.8
Measles in the past one year		
Yes	75	11.8
No	558	88.2
Child ever immunized		
Yes	547	86.4
No	86	13.6

^a^Breastfeeding

^b^Acute Respiratory Tract Infection

Nearly one-fourth (22.9 %) of the households used public tap water for consumption. Most of the households (78.5 %) took more than 30 min round trip to fetch water, while 60.8 % had no latrines (Table 3).

**Table 3:** Household related characteristics of parents, East Belesa District, northwest Ethiopia, 2014

Variables	Frequency	Percent
Source of drinking water		
River	138	21.8
Unprotected spring	120	19
Protected spring	230	36.3
Public tap	145	22.9
Water consumption per day		
<40 l	368	58.1
40–80 l	256	40.4
>80 l	9	1.5
Time to fetch water		
<15 min	29	4.6
15–30 min	107	16.9
>30 min	497	78.5
Water treatment by any means		
Yes	104	16.4
No	529	83.6
Method of waste disposal		
Open field disposal	446	70.5
Common pit	100	15.8
Other^a^	87	13.7
Latrine availability		
Yes	248	39.2
No	385	60.8
Type of house		
Thatched	535	84.5
Corrugated iron Sheet	98	15.5

^a^Burning and composting

### Prevalence of undernutrition

**Fig. 1:**
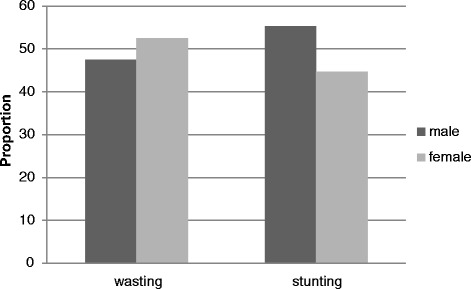
Distribution of stunting and wasting by sex among children aged 6–59 months, East Belesa District, northwest Ethiopia, 2014

**Fig. 2:**
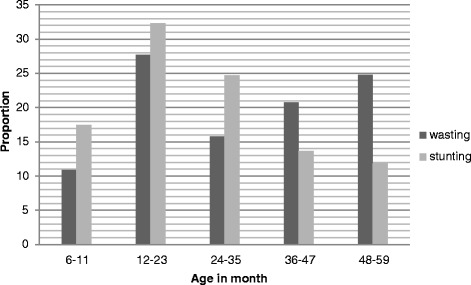
Distribution of stunting and wasting by age among children aged 6–59 months, East Belesa District, northwest Ethiopia, 2014

The overall prevalence of stunting and wasting among children aged 6–59 months was 57.7 % (95 % CI: 50.1, 65.2 %) and 16 % (95 % CI: 10.4, 21.6 %), respectively. Stunting was higher among male children and wasting among female ones (Fig. 1). However, both stunting and wasting were more prevalent among children aged 12–23 months (Fig. 2).

### Factors associated with undernutrition

In the bivariate analysis, child age, family monthly income, maternal antenatal care visit, family size, maternal age at first birth, and history of prelacteal feeding were significantly associated with stunting. However, the final multivariate logistic regression analysis revealed that advanced age of children (36–47 months) (AOR = 0.41; 95 % CI: 0.22, 0.78) and higher family monthly income, Et. Br. 750–1000 (AOR = 0.61; 95 % CI: 0.39, 0.92) and greater than Et. Br. 1000 (AOR = 0.45; 95 % CI: 0.23, 0.88) were significantly associated with reduced odds of stunting. Prelacteal feeding was associated with higher odds of stunting (AOR = 1.83; 95 % CI: 1.28, 2.61). Similarly, increased odds of stunting were observed among children whose mothers gave their first birth before 15 years of age compared to those whose mothers gave their first birth between 20 and 29 years of age (AOR = 2.47; 95 % CI: 1.19, 5.09) (Table 4).

**Table 4:** Factors associated with stunting among children aged 6–59 months, East Belesa District, northwest Ethiopia, 2014 (*n* = 633)

Variable	Stunting		COR ^**^(95%CI)	AOR^***^(95%CI)
	Yes	No		
Child age				
6–11	64	43	1	1
12–23	118	60	1.32 (0.81, 2.17)	1.15 (0.63, 2.11)
24–35	90	48	1.26 (0.75, 2.12)	1.21 (0.65, 2.27)
36–47	50	68	0.49 (0.29, 0.84)*	0.41 (0.22,0.78)*
48–59	43	49	0.59 (0.34, 1.04)	0.53 (0.27, 1.03)
Monthly income (in Et. Br.)				
<750	277	179	1	1
750–1000	66	68	0.62 (0.43, 0.92)*	0.61 (0.39, 0.92)*
>1000	22	21	0.67 (0.36, 1.26)	0.45 (0.23,0.88)*
Antenatal care follow-up				
No	271	180	1.41 (1.09, 1.99)*	
Yes	94	88	1	
Family size				
≥5	203	172	0.69 (0.50,0.96)	
<5	162	96	1	
Prelacteal feeding				
Yes	161	156	0.56 (0.41, 0.78)*	1.83 (1.28,2.61)*
No	204	112	1	1
Maternal age at first birth				
<15	48	15	3.00 (1.53,5.91)*	2.47 (1.19,5.09)*
15–19	252	192	1.23 (0.82,1.83)	1.12 (0.73, 1.73)
20–29	65	61	1	1

*significant at p of <0.05

**Crude Odds Ratio

***Adjusted Odds Ratio

The result of the bivariate analysis showed that child sex, vitamin-A supplementation in the past six months, type of prelacteal food given, and household water treatment were significantly associated with wasting. However, the final adjusted analysis revealed that the odds of wasting were 2.3 times higher among children who received butter as prelacteal food than those who received water (AOR = 2.32; 95 % CI: 1.82, 5.31) (Table 5).

**Table 5:** Factors associated with wasting among children aged 6–59 months, East Belesa District, northwest Ethiopia, 2014

Variables	Wasting		COR^**^(95%CI)	AOR ^***^95%CI
	Yes	No		
Child sex				
Male	48	291	0.75 (0.49, 0.95)^*^	
Female	53	241	1	
Child vitamin-A supplementation in the past six months				
Yes	77	352	1	
No	24	180	1.64 (1.00, 2.68)^*^	
Type of prelacteal food given (*n* = 370)				
Water	42	80	1	1
Butter	163	107	2.12 (1.47, 3.56)*	2.32 (1.81,5.31)*
Water treatment by any means				
Yes	11	93	1	
No	90	439	0.57 (0.29, 0.72)	

*significant at p of <0.05

**Crude Odds Ratio

***Adjusted Odds Ratio

## Discussion

According to WHO criteria, the public health significance of undernutrition is considered as very high/critical when the prevalence of stunting and wasting among children exceeds 40 and 15 %, respectively [32]. Accordingly, the prevalence of stunting (57.7 %) and wasting (16 %) in this study confirmed the very high public health significance of undernutrition in the study area. Advanced age of children and higher family monthly income were significantly associated with reduced odds of stunting. However, prelacteal feeding and giving the first birth before 15 years of age were associated with higher odds of stunting. In addition, taking butter as a prelacteal feed was positively associated with wasting.

The prevalence of stunting in this area was found to be the highest in comparison with other communities at home and abroad. For example, the prevalence in Gumbrit [6] and Gojjam [33], both in northwest Ethiopia, was 24 and 43.2 %, respectively, while Kenya [34], Vietnam [35], and Nepal [36] reported prevalence of 47, 20.7, and 41 %, respectively. The difference could be attributed to low agricultural productivity and repeated occurrence of drought which predisposes the community to recurrent food insecurity in our study area compared to the study areas in northwest Ethiopia. It could also be due to socio-economic and cultural situations of the study areas. It was also noted that food insecurity was associated with higher odds of stunting [36].

However, the prevalence of stunting in East Belesa was lower than the finding of a study in Afar Region, Ethiopia (67.8 %) [8]. The difference could be due to the livelihood of the people in Afar Region where agro-pastoral activities support the dwellers. Such a life style forces residents to live in temporal settlements which might pose difficulties for mothers to appropriately care for and feed their children throughout the year. In fact, poor child feeding practices [17, 20, 21, 37] and frequent infections [34] were the commonly reported predictors of undernutrition.

The prevalence of wasting in the study area was similar to reports from other Ethiopian settlements, like Oromia (16.8 %) [13], Tigray (11.6 %) [7], Gumbrit District (17.7 %) [6], Gojjam (14.8 %) [33], Afar Region (12.8 %) [8], and Bangladesh (Southeast Asia) (19.1 %) [38]. The magnitude of wasting in this study was also higher than the Ethiopian DHS report (2011) [1]. Another study in northwest Ethiopia [39], Kenya [34], and Nigeria [40] reported prevalence of 4.9, 2.6, and 3.7 %, respectively. This could be partially explained by the difference in socio-economic and food security status among the study areas. The prevalence was lower than what was reported from the Somali Region, Ethiopia (42.3 %) [30], urban slum of Ludhiana, India (42 %) [41], and Chitwan District, Nepal (25.7 %) [42].

In this study, a higher family monthly income was associated with lower odds of stunting. Similar findings were reported elsewhere [6, 18, 43]. This could be due to the fact that low income might result in loss of the household purchasing power to procure nutritious and diversified food. This may increase the likelihood of infection through such mechanisms as inadequate personal and environmental hygiene. It was noted that recurrent infections and poor personal and environmental hygiene were significant determinants of undernutrition [44].

Children aged 36–47 months were less likely to be stunted compared with infants aged 6–11 months. This could be due to the fact that the latter have poorer nutritional reserve capacity compared to the former. Poor nutritional reserve capacity with chronic exposure to poor quality complementary food increases the child’s risk of developing stunting. Other studies also claimed that infancy was a component of the most critical period for the risk of stunting and its irreversible damage [1, 43].

The odds of stunting among children born to mothers who gave their first birth before 15 years of age were high. Adolescence, a transition period between childhood and adulthood, is a time of rapid growth and development, demanding extra energy and micronutrients. Nevertheless, in case of early pregnancy (before 15 years of age), the mother and the growing fetus compete for nutrients to support their rapid growth. As a result, pregnancy during adolescence slows down the girl’s growth [45]. However, low maternal stature was independently associated with Intra-Uterine Fetal Growth Restriction (IUGR) and childhood stunting [3].

Children given prelacteal feeds were more likely to be stunted than their counterparts. The finding was consistent with a previous report from Gojjam, northwest Ethiopia [33]. Similarly, children who were given butter as prelacteal food were at a higher risk of wasting as compared to those who took water. This finding was supported by a report from the Somali Region, Ethiopia [30]. This could be explained by the fact that prelacteal food impairs the newborns’ opportunity to obtain immune factors through feeding on colostrum [46] and increases the risk of the inoculation of pathogenic microorganisms through contaminated prelacteal food. The pathogenic microorganisms might enhance host susceptibility to different infectious diseases and cause damage to the intestinal mucosal cells, thereby leading to malabsorption of nutrients [45].

Although a thorough attempt has been made to show the current burden of undernutrition in this drought prone area, the study is not free from certain limitations. Firstly, the cross-sectional nature of the study design neither represents the seasonal variations of nutritional outcomes, particularly that of wasting status nor establishes the correct temporal causal relationships of the predictors and the outcome variables. Secondly, the study is not free from measurement errors in gathering anthropometric data although a pretest, instrument calibration, and close supervisions were carried out to minimize bias.

## Conclusion

In this community, child undernutrition is a critical public health problem. Advanced age (36–47 months) of children and higher family monthly income were significantly associated with lower odds of stunting. However, higher odds of stunting were observed among mothers who gave their first birth before 15 years of age and children who received prelacteal feeds. The likelihood of developing wasting increased among children who were given butter as prelacteal food. Thus, efforts should be intensified to further improve the socio-economic status of the community. Moreover, delaying mothers’ first pregnancy and reducing prelacteal feeding is of a paramount significance in reducing the burden of undernutrition.

## Abbreviations

AOR, adjusted odds ratio; CI, confidence interval; COR, crude odds ratio; DHS, Demographic and Health Survey; GDP, gross domestic product; HAZ, Hight-for-Age Z-score; WHO, World Health Organization; WHZ, Weight-for-Hight Z-score
